# Altered Circulating Biomarkers of Purinergic Signaling, Inflammasome-Related Pathways, Pyroptosis, and Oxidative Stress in Fibromyalgia

**DOI:** 10.3390/ijms27156579

**Published:** 2026-07-24

**Authors:** Emrullah Hayta, Tugba Agbektas, Gonca Kabak, Gokhan Dogan, Ayca Tas, Yavuz Silig

**Affiliations:** 1 Department of Physical Therapy and Rehabilitation, Ataşehir Acibadem Hospital, Acibadem University, Istanbul 34758, Türkiye; dremay@gmail.com; 2 Department of Food Processing Technologies Services, Yıldızeli Vocational School, Sivas Cumhuriyet University, Sivas 58140, Türkiye; tubaagbektas@cumhuriyet.edu.tr; 3 Department of Biochemistry, Faculty of Medicine, Sivas Cumhuriyet University, Sivas 58140, Türkiye; gonca.kabak24@gmail.com (G.K.); gokhandogan@cumhuriyet.edu.tr (G.D.); ysilig@cumhuriyet.edu.tr (Y.S.)

**Keywords:** fibromyalgia, PANX1, P2RX7, NLRP3 inflammasome, pyroptosis, caspase-1, interleukin-1β, interleukin-18, gasdermin D, oxidative stress

## Abstract

(1) Fibromyalgia syndrome (FMS) is a chronic pain disorder with a multifactorial pathogenesis involving neuroinflammation, oxidative stress, and immune dysfunction. This study aimed to evaluate serum biomarkers related to purinergic signaling, inflammasome activation, pyroptosis, and oxidative stress in patients with FMS. (2) Methods: A total of 93 patients with FMS and 93 age- and sex-matched healthy controls were enrolled. Serum levels of pannexin-1 (PANX1), purinergic receptor P2X7 (P2RX7), NLRP3, caspase-1 (CASP1), interleukin-1β (IL-1β), interleukin-18 (IL-18), gasdermin D (GSDMD), and gasdermin E (GSDME) were measured using enzyme-linked immunosorbent assays (ELISA). (3) Results: Total antioxidant status (TAS) and total oxidant status (TOS) were determined. Patients with FMS exhibited significantly increased serum CASP1, GSDME, PANX1, P2RX7, and total oxidative stress (TOS) levels, whereas GSDMD, NLRP3, IL-18, and TAS levels were significantly decreased compared with controls (all *p* < 0.05). No significant differences were observed in IL-1β levels. Receiver operating characteristic analysis demonstrated exploratory discriminatory performance within this case–control cohort for CASP1 and TAS (AUC = 0.898), followed by TOS (AUC = 0.849) and P2RX7 (AUC = 0.757). (4) Conclusions: These findings indicate alterations in circulating biomarkers related to purinergic signaling, inflammasome-associated pathways, pyroptosis-related proteins, and oxidative stress in patients with FMS. These alterations may contribute to the pathophysiology of FMS and provide a foundation for future mechanistic studies investigating their potential as biomarkers and therapeutic targets.

## 1. Introduction

Fibromyalgia syndrome (FMS) is a chronic pain syndrome characterized by widespread musculoskeletal pain, chronic fatigue, sleep disturbance, cognitive dysfunction, and psychological symptoms. This disorder is more prevalent among women and significantly affects their daily activities and quality of life. Although fibromyalgia was long considered to result solely from altered pain perception within the central nervous system, it is now recognized as a complex disorder with a multifactorial pathophysiology. Recent studies have suggested that immune system activation, neuroinflammation, oxidative stress, mitochondrial dysfunction, and inflammatory cell death mechanisms may play important roles in the development and progression of fibromyalgia [1,2]. Chronic inflammation is believed to contribute to disease persistence, and an interaction between peripheral inflammatory responses and central sensitization has been proposed [2,3]. Central sensitization is considered one of the fundamental mechanism underlying the pathogenesis of fibromyalgia. During this process, hypersensitivity develops within the pain transmission pathways in the spinal cord and brain, resulting in a lowered pain threshold and the perception of normally non-painful stimuli as painful. This amplification of pain signaling has been associated with both neuronal mechanisms and neuroimmune processes. Microglial activation, increased production of pro-inflammatory cytokines, and glial–neuron interactions contribute to the maintenance of chronic pain. Therefore, fibromyalgia is currently regarded not only as a neurological disorder but also as a systemic disease with significant immunological components [3,4].

Among the inflammatory mechanisms associated with fibromyalgia, inflammasome complexes have recently attracted considerable attention. Inflammasomes are cytoplasmic multiprotein complexes that detect cellular stress and damage signals, subsequently initiating inflammatory responses. Among these complexes, the NLRP3 inflammasome is one of the most extensively investigated. The NLRP3 inflammasome can be activated by various cellular danger signals, including reactive oxygen species (ROS), ATP release, mitochondrial damage, ionic imbalance, and lysosomal stress [5]. Following activation, caspase-1 is activated, leading to the conversion of the proinflammatory cytokines interleukin-1β (IL-1β) and interleukin-18 (IL-18) into their biologically active forms. This process ultimately results in a potent immune response that amplifies inflammation [5,6]. Several studies have reported increased levels of NLRP3 inflammasome-related molecules and inflammatory cytokines in patients with fibromyalgia. IL-1β enhances neuronal excitability, facilitates synaptic transmission, and promotes central sensitization. Furthermore, IL-1β activates microglial cells, thereby increasing neuroinflammation at the spinal cord level [6,7]. IL-18 is thought to enhance immune system activation and contribute to the persistence of chronic inflammation. Therefore, elevated levels of these cytokines may be associated with pain, fatigue, and depressive symptoms commonly observed in patients with fibromyalgia [7]. Another important component involved in inflammasome activation is the P2RX7 receptor. This ATP-sensitive purinergic receptor is activated in response to tissue injury and cellular stress, resulting in potassium efflux and subsequent activation of the NLRP3 inflammasome [8]. The P2RX7 receptor is highly expressed in macrophages and microglial cells, and its prolonged activation is associated with increased ROS production, mitochondrial dysfunction, and enhanced inflammatory signaling. In addition, pannexin-1 (PANX1) channels facilitate extracellular ATP release, thereby promoting P2RX7 activation and sustaining the inflammatory process [9]. Therefore, ATP-mediated purinergic signaling has been proposed to contribute to the mechanisms of chronic pain in fibromyalgia [8,9].

Activation of the NLRP3 inflammasome not only increases the production of inflammatory cytokines but may also initiate pyroptosis, an inflammatory form of programmed cell death. During this process, gasdermin D (GSDMD) is cleaved by activated caspase-1, resulting in the formation of pores in the cell membrane. Disruption of membrane integrity subsequently leads to the release of intracellular inflammatory contents into the extracellular environment, thereby exacerbating inflammation [10]. In addition, secondary pyroptotic mechanisms mediated by gasdermin E (GSDME) have been reported to participate in chronic inflammatory processes [11]. Inflammatory mediators and damage-associated molecular patterns released during pyroptosis can further amplify inflammatory responses in neighboring cells, thereby contributing to the maintenance of the chronic pain cycle [10,11]. Taken together, these findings suggest that the PANX1–P2RX7–NLRP3 inflammasome axis may play an important role in the pathogenesis of fibromyalgia through its involvement in inflammation, neuroinflammation, and pyroptotic cell death. Persistent but low-grade activation of these molecular pathways may increase pain sensitivity within the central nervous system and contribute to the chronicity of symptoms [12]. Furthermore, the interactions among oxidative stress, mitochondrial dysfunction, and inflammatory cell death mechanisms may constitute the biological basis for chronic pain in fibromyalgia [13]. Currently, the NLRP3 inflammasome, P2RX7 receptor, PANX1 channels, and gasdermin proteins are considered potential biomarkers and therapeutic targets for the development of future targeted treatment strategies.

Despite increasing evidence implicating neuroinflammation, oxidative stress, and immune dysregulation in fibromyalgia, the contributions of purinergic signaling, inflammasome activation, and pyroptosis-related pathways to disease pathogenesis remain unclear. In particular, the potential involvement of the PANX1–P2RX7–NLRP3 signaling axis and gasdermin-mediated inflammatory cell death mechanisms has not been comprehensively investigated in patients with fibromyalgia. Therefore, the present study aimed to evaluate serum levels of PANX1, P2RX7, NLRP3, CASP1, IL-1β, IL-18, GSDMD, and GSDME in patients with fibromyalgia and healthy controls. We also assessed total antioxidant status (TAS) and total oxidant status (TOS) to explore the relationship between oxidative stress and inflammasome-associated inflammatory responses. By examining these interconnected molecular pathways, this study aimed to provide novel insights into the mechanisms underlying fibromyalgia and identify potential biomarkers associated with chronic pain and disease pathogenesis.

## 2. Results

The study included 93 patients with FMS and 93 healthy controls. The distribution of gender, age, and occupational status was comparable between the groups, with no statistically significant differences observed for gender, age, or occupation (*p* = 0.561, *p* = 0.165, and *p* = 0.245, respectively). In contrast, several clinical symptoms were significantly more frequent in the FMS group than in the control group. Sleep disturbance, fatigue, headache, morning fatigue, dry mouth, leg numbness, dry eyes, concentration difficulty, soft tissue swelling sensation, and a family history of fibromyalgia were significantly more common among patients with FMS (*p* < 0.05 for all) (Table 1).

Serum protein levels differed significantly between the patient and control groups for most evaluated biomarkers. CASP1, GSDME, PANX1, P2RX7, and TOS levels were significantly higher in patients with FMS than in healthy controls, whereas GSDMD, NLRP3, IL18, and TAS levels were significantly lower in the FMS group. No statistically significant difference was observed in IL1B levels between the groups (*p* = 0.248). These findings indicate marked alterations in inflammasome/pyroptosis-related markers, purinergic signaling-associated proteins, and oxidative stress parameters in patients with FMS (Table 2).

**Figure 1:**
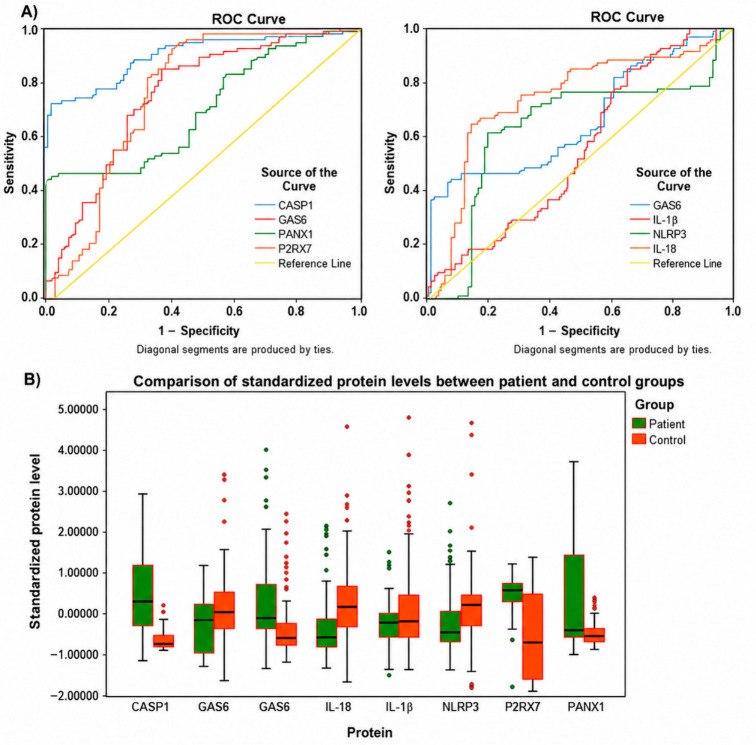
(**A**) ROC curve analyses illustrating the discriminative performance of the evaluated protein markers between the patient and control groups. (**B**) Distribution of standardized protein levels in patient and control groups, presented as box plots.

**Figure 2:**
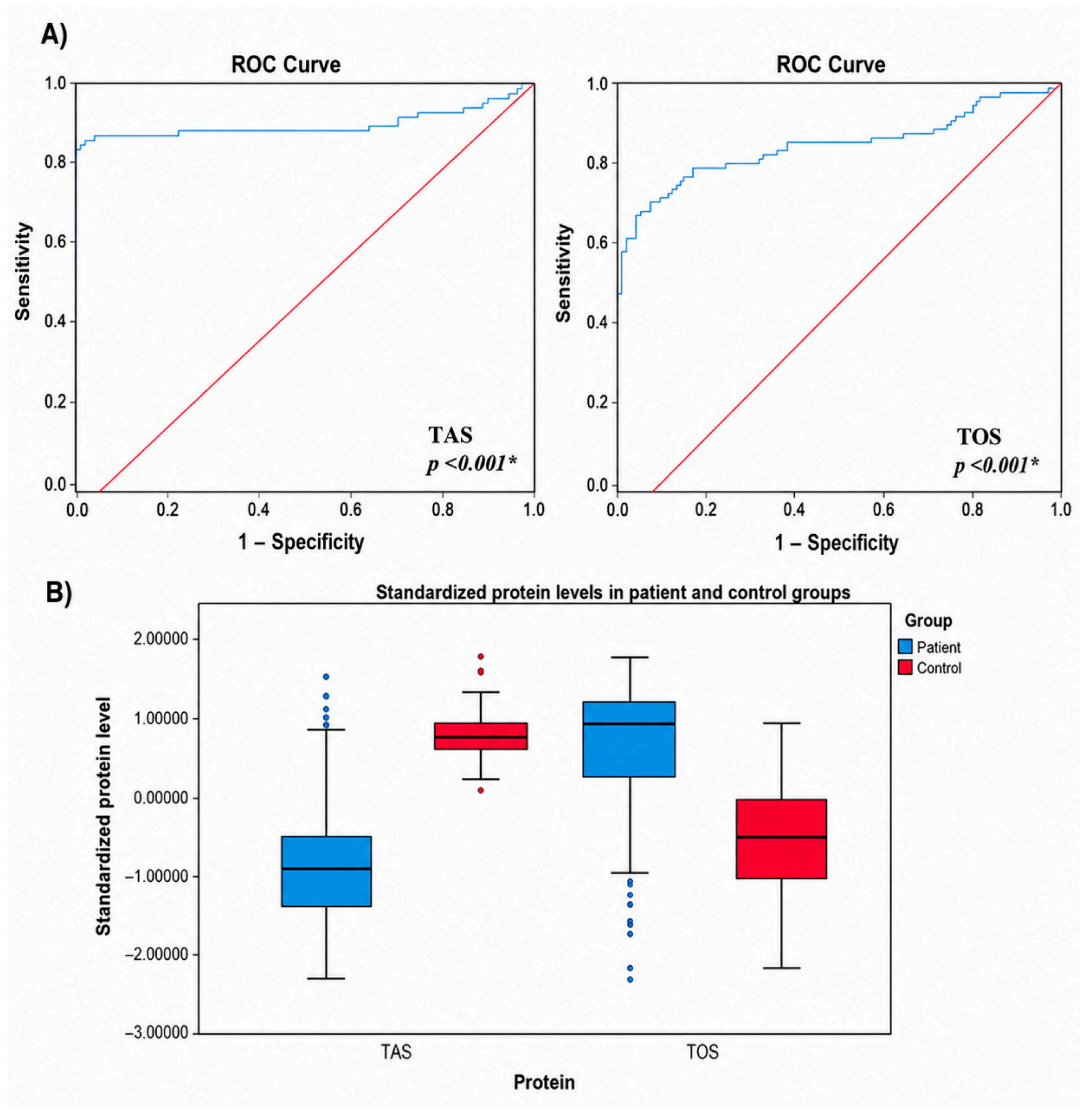
(**A**) ROC curve analyses demonstrating the discriminative performance of TAS and TOS between the patient (blue) and control groups (red). (**B**) Box plot analysis showing the distribution of standardized TAS and TOS levels in patient and control groups.

ROC curve analysis demonstrated that several serum biomarkers showed discriminatory ability between patients with FMS and healthy controls within this case–control sample. CASP1 and TAS yielded the highest AUC values (0.898 each), followed by TOS (AUC = 0.849), P2RX7 (AUC = 0.757), GSDME (AUC = 0.754), IL18 (AUC = 0.735), PANX1 (AUC = 0.704), GSDMD (AUC = 0.655), and NLRP3 (AUC = 0.626). IL1B did not significantly discriminate between the groups (*p* = 0.248). The ROC curves and standardized box plot distributions further illustrated the differing biomarker profiles observed between patients and controls (Table 3, Figure 1 and Figure 2). These findings reflect discrimination within the present study sample and should not be interpreted as evidence of established clinical diagnostic utility without external validation. A Bonferroni-adjusted significance threshold of *p* < 0.005 was applied for the ROC analyses of 10 biomarkers; all biomarkers with nominally significant ROC results remained significant after correction, whereas IL1B remained non-significant.

Given the number of biomarker comparisons and subsequent ROC, regression, and correlation analyses, the regression and correlation analyses were considered exploratory. Therefore, nominal *p* values are reported for these analyses, and findings with *p* values close to 0.05 should be interpreted cautiously.

Binary logistic regression analysis was performed to identify the clinical parameters independently associated with FMS. Headache, morning fatigue, dry mouth, leg numbness, and difficulty concentrating were significantly associated with the presence of FMS. Among these variables, concentration difficulty showed the strongest association with FMS (OR = 6.052, 95% CI: 2.386–15.349, *p* < 0.001), followed by morning fatigue (OR = 4.644, 95% CI: 1.598–13.499, *p* = 0.005), leg numbness (OR = 2.902, 95% CI: 1.251–6.732, *p* = 0.013), headache (OR = 2.813, 95% CI: 1.121–7.056, *p* = 0.028), and dry mouth (OR = 2.741, 95% CI: 1.136–6.615, *p* = 0.025). Sleep disturbance, fatigue, dry eyes, and soft tissue swelling were not independently significant in the multivariable model (Table 4).

Pathway-based binary logistic regression models were constructed to evaluate the independent associations of protein biomarkers with FMS status after adjustment for age. In the purinergic signaling model, higher PANX1 and P2RX7 levels were independently associated with an increased likelihood of FMS (*p* < 0.001 for both). In the inflammasome model, higher CASP1 levels remained independently associated with FMS (OR = 2.261, 95% CI: 1.737–2.944, *p* < 0.001), whereas NLRP3 and IL18 were not significant after adjustment. In the pyroptosis executioner model, higher GSDME levels were associated with an increased FMS likelihood, while higher GSDMD levels were associated with a decreased FMS likelihood (*p* < 0.001 and *p* = 0.004, respectively). In the oxidative stress model, lower TAS and higher TOS levels were independently associated with FMS status (*p* < 0.001 for both), indicating a strong association between impaired oxidative balance and FMS (Table 5).

Correlation analysis using Spearman’s rank correlation coefficient revealed several significant associations between clinical characteristics and serum biomarkers in the overall study population. Sleep disturbance was negatively correlated with CASP1, GSDME, P2RX7, and TOS levels, whereas it was positively correlated with TAS levels. Morning fatigue was negatively associated with CASP1 and TOS and positively associated with TAS levels. Similarly, dry mouth was negatively correlated with CASP1, P2RX7, and TOS, while showing positive correlations with IL18 and TAS. Concentration difficulty was negatively correlated with CASP1 and TOS levels and positively correlated with TAS levels. Soft tissue swelling sensation was negatively correlated with CASP1, GSDMD, and P2RX7 levels. In addition, a family history of fibromyalgia was negatively correlated with CASP1, PANX1, P2RX7, GSDME, and TOS and positively correlated with GSDMD, NLRP3, IL18, and TAS levels. These findings suggest that clinical symptom patterns may be associated with alterations in inflammasome-related, purinergic signaling, pyroptosis-related, and oxidative stress biomarkers (Table 6).

To explore whether self-reported sicca-related symptoms were associated with circulating biomarker levels independently of FMS status, additional subgroup analyses were performed within the healthy control group. No significant differences were observed in CASP1, GSDMD, GSDME, IL1B, PANX1, P2RX7, NLRP3, IL18, TAS, or TOS levels between controls with and without self-reported dry eye symptoms. Similarly, no significant differences were detected according to the presence of self-reported dry mouth symptoms (all *p* ≥ 0.05) (Table 7).

## 3. Discussion

Fibromyalgia syndrome (FMS) is increasingly recognized as a complex disorder involving neuroimmune dysregulation, neuroinflammation, mitochondrial dysfunction, oxidative stress, and central sensitization mechanisms [1,2,3,4]. In the present study, patients with FMS exhibited significantly elevated serum PANX1, P2RX7, CASP1, GSDME, and TOS levels, whereas those of TAS, NLRP3, IL-18, and GSDMD were significantly reduced. Collectively, these findings indicate alterations in circulating biomarkers related to purinergic signaling, inflammasome-associated pathways, pyroptosis-related proteins, and oxidative stress in patients with FMS. These alterations may be associated with disease pathophysiology but should not be interpreted as direct evidence of functional pathway activation.

One of the most important findings of this study was the significant elevation of PANX1 and P2RX7 levels in patients with FMS. Pannexin-1 channels represent a major pathway for ATP release from stressed or damaged cells, whereas P2RX7 is an ATP-gated receptor that plays a central role in inflammatory signaling and chronic pain development [8,14,15,16]. Activation of PANX1 channels promotes extracellular ATP accumulation, which subsequently activates P2RX7 receptors and initiates downstream inflammatory cascades. Experimental evidence has demonstrated that activation of the PANX1–P2RX7 axis contributes to microglial activation, cytokine release, and hypersensitivity to pain [15,16]. Furthermore, D’Amico et al. showed that pharmacological inhibition of P2RX7 significantly attenuated fibromyalgia-like symptoms and suppressed NLRP3 inflammasome activation in an experimental FMS model [15]. Therefore, the elevated circulating PANX1 and P2RX7 protein levels observed in the present study may reflect alterations in biomarkers associated with ATP-mediated purinergic signaling. However, because functional assays were not performed, these findings should not be interpreted as direct evidence of enhanced pathway activation.

Growing evidence indicates that microglial activation contributes significantly to chronic pain and central sensitization. Activated microglia release ATP, reactive oxygen species (ROS), IL-1β, TNF-α, and other inflammatory mediators that enhance neuronal excitability and maintain chronic pain states [4,17]. Neuroimaging studies have also demonstrated increased neuroinflammation in several brain regions in patients with fibromyalgia [18]. Consequently, the increased circulating PANX1 and P2RX7 concentrations observed in our study may be consistent with alterations in biomarkers previously associated with neuroimmune processes. Nevertheless, serum protein concentrations alone cannot establish ongoing neuroimmune activation.

Another notable finding was the marked increase in CASP1 levels. Caspase-1 is a central effector molecule responsible for the maturation of IL-1β and IL-18 and is considered a hallmark of inflammasome activation [19]. Elevated circulating CASP1 levels observed in our patients are consistent with alterations in biomarkers related to innate immune responses; however, they do not directly demonstrate functional inflammasome activation. In contrast to the reduced circulating NLRP3 levels observed in our study, Cordero et al. reported NLRP3 inflammasome activation and increased inflammatory mediators in patients with fibromyalgia [14]. This discrepancy may reflect differences in biological specimens, study populations, disease heterogeneity, or the distinction between circulating protein concentrations and intracellular inflammasome activity. Because only circulating CASP1 protein concentrations were measured, the functional activity of caspase-1 could not be determined. Future studies evaluating enzymatic activity are needed to clarify its contribution to chronic pain.

Interestingly, despite elevated CASP1 levels, NLRP3 and IL-18 concentrations were significantly lower in the FMS group. At first glance, this pattern appears inconsistent with a uniformly activated canonical inflammasome response. Several potential explanations may account for this discordant profile, including differential regulation of circulating proteins, feedback-related changes, altered protein turnover, timing-dependent cytokine release, and differences between systemic circulation and tissue-specific inflammatory activity [12,19]. Importantly, these mechanisms were not directly evaluated in the present study. Serum biomarker levels may also not accurately reflect tissue-specific or central nervous system inflammasome activity. Previous studies have suggested that neuroinflammation, rather than systemic inflammation, may play a prominent role in fibromyalgia, which could partly explain discrepancies between circulating and tissue-associated inflammatory markers [18]. Importantly, the overall biomarker profile observed in the present study does not support coordinated activation of the canonical PANX1–P2RX7–NLRP3 inflammasome pathway. Although circulating PANX1, P2RX7, and CASP1 concentrations were increased, NLRP3 and IL-18 levels were reduced, whereas IL-1β remained unchanged. Therefore, these findings should be interpreted as alterations in circulating pathway-related biomarkers rather than direct evidence of canonical inflammasome activation or pyroptosis. Alternative explanations, including differential regulation of circulating proteins, compartment-specific inflammatory responses, and the inherent limitations of serum-based biomarker assessment, should also be considered.

The pyroptosis-related findings of this study are also noteworthy. While GSDME levels were significantly elevated, GSDMD levels were reduced in patients with FMS. Gasdermin D is traditionally regarded as the principal executor of canonical inflammasome-mediated pyroptosis, whereas GSDME is associated with alternative inflammatory cell death pathways triggered by caspase activation [10,11]. Current evidence indicates that the activation of different members of the gasdermin family does not occur through a uniform mechanism. Rather, it is a selective process governed by the cellular microenvironment, the degree of chronic inflammation, and the disease-specific molecular context. Therefore, increased circulating GSDME concentrations may reflect alterations in biomarkers associated with inflammatory cell death pathways. However, because gasdermin cleavage and pore formation were not evaluated, functional pyroptosis cannot be confirmed. This interpretation is supported by studies demonstrating the complex interactions among caspases, gasdermins, and inflammatory signaling pathways during chronic inflammatory disorders [10,19].

Oxidative stress is another key mechanism involved in the pathogenesis of fibromyalgia. In the present study, TOS levels were significantly elevated, whereas TAS levels were markedly reduced, indicating a pronounced shift toward oxidative imbalance. These findings are consistent with previous studies reporting increased ROS production, mitochondrial dysfunction, lipid peroxidation, and impaired antioxidant defense systems in patients with FMS [13,20]. Oxidative stress has been shown to directly activate inflammatory pathways, including the NLRP3 inflammasome, establishing a vicious cycle between oxidative damage and inflammation [14,20]. Cordero et al. proposed that mitochondrial dysfunction and oxidative stress may act as upstream triggers of inflammasome activation in fibromyalgia [13,14]. Therefore, the observed TAS and TOS alterations may reflect oxidative imbalance associated with FMS. Whether oxidative stress directly contributes to activation of purinergic or inflammasome-related pathways requires further functional investigation.

ROC analyses showed that CASP1 and TAS had the highest discriminatory ability between patients with FMS and healthy controls, followed by TOS and P2RX7. These findings suggest that circulating biomarkers related to inflammasome-associated pathways and oxidative stress may have exploratory value for distinguishing the groups within the present case–control sample. However, because the study lacked external validation and used a case–control design, these results should not be interpreted as evidence of established clinical diagnostic utility. No single biomarker can currently be recommended for the routine diagnosis of fibromyalgia. Nevertheless, these findings may warrant further investigation as part of broader biomarker panels for disease characterization and the exploration of biologically distinct patient subgroups.

Interestingly, dry eye and dry mouth symptoms were associated with selected circulating biomarkers in the present study. Similar sicca-related symptoms are commonly observed in chronic inflammatory disorders, particularly dry eye disease and Sjögren’s syndrome, where persistent immune activation, oxidative stress, and inflammasome-associated inflammatory responses may contribute to disease pathogenesis. Recent evidence has demonstrated that NLRP3 inflammasome-related signaling may play a role in ocular surface inflammation in dry eye disease. Increased expression of NLRP3, caspase-1, IL-1β, and IL-18 has also been reported in tears and ocular surface samples from patients with dry eye disease, particularly Sjögren’s syndrome-associated dry eye [21]. Although dry eye and dry mouth in the present study were recorded as patient-reported symptoms according to the 2016 ACR Fibromyalgia Diagnostic Criteria rather than objective ophthalmological or salivary gland function tests, the observed associations with circulating biomarkers may suggest partially shared inflammatory and oxidative mechanisms underlying sicca-related symptoms across chronic inflammatory conditions. To further explore whether sicca-related symptoms alone might influence circulating biomarker levels, additional exploratory subgroup analyses were performed within the healthy control group. No significant differences were observed in CASP1, GSDMD, GSDME, IL1B, PANX1, P2RX7, NLRP3, IL18, TAS, or TOS levels between controls with and without self-reported dry eye or dry mouth symptoms.

These findings suggest that the biomarker differences observed between patients with FMS and healthy controls are unlikely to be explained solely by self-reported sicca-related symptoms. However, this interpretation remains limited because dry eye and dry mouth were not confirmed using objective ophthalmological or salivary gland assessments. Future studies incorporating such assessments are warranted to better define these relationships [22].

## 4. Materials and Methods

### 4.1. Study Population

Patients included in the study were recruited from individuals presenting to the Department of Physical Therapy and Rehabilitation at Sivas Cumhuriyet University Health Services Practice and Research Hospital, Sivas, Türkiye. The serum samples used in the present study were originally collected under ethics approval granted by Sivas Cumhuriyet University Local Ethics Committee (Approval No. 2015-05/01, Date: 12 May 2015) as part of a previous fibromyalgia research project. The current study represents a secondary analysis of these archived serum samples. Accordingly, an additional ethics approval for the reuse of archived biological materials and the present analyses was obtained from Sivas Cumhuriyet University Non-Interventional Clinical Research Ethics Committee (Approval No. 2026-05/44, Date: 07 May 2026). The study was conducted in accordance with the Declaration of Helsinki, and written informed consent was obtained from all participants at the time of the original sample collection. The fibromyalgia (FM) group consisted of 93 patients with an established diagnosis of fibromyalgia according to the 2016 American College of Rheumatology (ACR) classification criteria, who had experienced chronic widespread pain for at least three months. Clinical characteristics were collected using a standardized clinical assessment based on the 2016 ACR Fibromyalgia Diagnostic Criteria. Accordingly, symptoms such as dry eye, dry mouth, sleep disturbance, fatigue, headache, morning fatigue, leg numbness, concentration difficulty, and soft tissue swelling sensation were recorded from patient-reported responses obtained during the clinical evaluation. The control group comprised 93 age- and sex-matched healthy volunteers who attended the same institution and had no history of chronic pain syndromes or other significant medical conditions. The patient and control groups were randomly selected to ensure comparability with respect to age and sex of the participants. The exclusion criteria were as follows: age <18 or >65 years, refusal to provide informed consent, and the presence of cancer, autoimmune diseases, endocrine disorders, severe hepatic or renal insufficiency, active infections, inflammatory or neurodegenerative disorders, or major psychiatric illnesses. Individuals who were pregnant or breastfeeding, had undergone major surgery within the previous three months, or had received corticosteroid, immunosuppressive, biologic, or opioid therapy during the preceding three months were also excluded from the study. Furthermore, participants who had received any medical treatment within the previous month, those with menstrual irregularities, perimenopausal symptoms (including menstrual cycle irregularities, hot flashes, night sweats, and vaginal dryness), postmenopausal women, individuals with hemolyzed or inadequate serum samples, and those with incomplete clinical data were excluded from the study. Perimenopausal and postmenopausal women were excluded to minimize the potential influence of menopause-related hormonal alterations on inflammatory, oxidative stress, and pyroptosis-associated biomarkers. The healthy control group was recruited from individuals presenting to the same department who had not received any clinical diagnosis and were selected to be comparable to the patient group in terms of age and sex.

The present study was conducted using archived serum samples collected as part of a previously approved research project, and no additional blood samples were obtained from the participants.

### 4.2. Sample Collection

Blood samples were centrifuged at 3000 rpm for 10 min to separate the serum phase and were immediately stored at −80 °C until biochemical analyses were performed. The patient group consisted of individuals previously diagnosed with fibromyalgia, while serum samples from healthy individuals in the control group were collected during the same period and archived in accordance with the ethics committee approval. All serum samples were processed, stored, and analyzed under identical conditions and underwent a single freeze–thaw cycle prior to ELISA analysis.

### 4.3. Protein Level Assay

Serum concentrations of CASP1 (Cat. No. E2248Hu), GSDMD (Cat. No. E6838Hu), GSDME (Cat. No. E6841Hu), IL-1β (Cat. No. E0143Hu), IL-18 (Cat. No. E0147Hu), PANX1 (Cat. No. E4270Hu), P2RX7 (Cat. No. E5825Hu), and NLRP3 (Cat. No. E3886Hu) were determined using commercially available ELISA kits (Bioassay Technology Laboratory, Shanghai, China) according to the manufacturer’s instructions. The assay ranges and sensitivities of the kits were 0.13–8 ng/mL and 0.06 ng/mL for PANX1, 15–3000 ng/L and 7.16 ng/L for P2RX7, 2–600 pg/mL and 1.06 pg/mL for NLRP3, 0.1–38 ng/mL and 0.051 ng/mL for CASP1, 20–6000 pg/L and 10.07 pg/L for IL-1β, 0.5–100 ng/L and 0.2 ng/L for IL-18, 0.05–15 ng/mL and 0.017 ng/mL for GSDMD, and 0.05–15 ng/mL and 0.016 ng/mL for GSDME, respectively. For all assays, the intra-assay coefficient of variation (CV) was <8%, and the inter-assay CV was <10%.

For the ELISA procedure, 40 µL of sample diluent and 10 µL of serum sample were added to each well. The plates were incubated at 37 °C for 30 min. Subsequently, the wells were washed five times with wash buffer, and horseradish peroxidase (HRP)-conjugated reagent was added. The plates were then incubated at 37 °C for 30 min. After a second wash, chromogenic substrates were added and incubated in the dark for 15 min. The reaction was terminated with a stop solution, and the optical density was measured at 450 nm using a microplate reader. Protein concentrations were quantitatively calculated from the absorbance values using standard calibration curves. All analyses were performed according to the manufacturer’s recommended protocols. All serum samples were analyzed in duplicate, and the mean value of the two measurements was used for statistical analysis. All serum samples were coded before laboratory analysis, and the personnel performing the ELISA measurements were blinded to the participants’ clinical group allocation throughout the analytical procedures.

### 4.4. Analysis of Total Antioxidant and Oxidant Levels

Serum total antioxidant status (TAS) and total oxidant status (TOS) were determined using commercially available Rel Assay Diagnostics kits (RL0017 and RL0024, Rel Assay Diagnostics, Gaziantep, Türkiye) according to the manufacturer’s instructions [23,24,25]. The TAS assay is based on the inhibition of ABTS radical cation formation by antioxidants present in the sample, with the resulting decrease in absorbance being proportional to the total antioxidant capacity. TAS results were expressed as mmol Trolox equivalent/L. According to the manufacturer, the analytical measurement range of the assay was 0.01–4.00 mmol Trolox equivalent/L.

The TOS assay is based on the oxidation of ferrous ions to ferric ions by oxidant molecules present in the sample. The ferric ions subsequently form a colored complex with xylenol orange, which is measured spectrophotometrically. The assay was calibrated using hydrogen peroxide, and the results were expressed as μmol H_2_O_2_ equivalent/L. According to the manufacturer, the analytical measurement range of the assay was 0.2–80 μmol H_2_O_2_ equivalent/L. All serum samples were analyzed without additional dilution in accordance with the manufacturer’s protocol. For statistical analyses, TAS and TOS values were additionally standardized using Z-score normalization. However, the descriptive statistics presented in Table 2 are reported as the original calculated concentrations, expressed as mmol Trolox equivalent/L for TAS and μmol H_2_O_2_ equivalent/L for TOS. No dilution-based recalculation was performed.

### 4.5. Statistical Analysis

All statistical analyses were performed using IBM SPSS Statistics version 23.0 (IBM Corp., Armonk, NY, USA). Continuous variables were assessed for distributional characteristics before statistical testing. Normally distributed continuous variables were summarized as the mean ± standard deviation, whereas non-normally distributed biomarker data were presented the as median (Q1–Q3) and minimum–maximum values. Categorical variables were expressed as frequency and percentage. Age was compared between patients with fibromyalgia syndrome (FMS) and healthy controls using the independent-samples *t*-test. Non-normally distributed serum biomarker levels were compared using the Mann–Whitney U test. Categorical variables, including sex, occupation, sleep disturbance, fatigue, headache, morning fatigue, dry mouth, leg numbness, dry eyes, concentration difficulty, soft tissue swelling sensation, and family history of fibromyalgia, were analyzed using the chi-square test or Fisher’s exact test when expected cell counts were low. Binary logistic regression analysis was performed to identify clinical factors independently associated with FMS. Regression results were reported as beta coefficients, standard errors, odds ratios [Exp(B)], and 95% confidence intervals. Additional pathway-based binary logistic regression models were constructed to assess biomarker-related associations according to biological relevance and were adjusted for age. Receiver operating characteristic (ROC) curve analysis was used to assess the discriminatory ability of serum biomarkers for distinguishing patients with FMS from healthy controls within the present case–control sample. The area under the curve, 95% confidence interval, optimal cut-off value, sensitivity, and specificity were calculated. Optimal cut-off values were determined using the Youden index. Positive and negative predictive values were not reported because they are influenced by the artificial disease prevalence inherent to the case–control design. A Bonferroni-adjusted significance threshold of *p* < 0.005 was applied for ROC analyses of the 10 biomarkers. Spearman’s rank correlation analysis was used to assess associations between sociodemographic characteristics, clinical findings, and biomarker levels because several biomarkers were non-normally distributed and several clinical variables were binary. Correlation coefficients were presented with corresponding two-tailed *p* values. Regression and correlation analyses were considered exploratory because of the number of comparisons performed; therefore, nominal *p* values are reported, and marginal findings were interpreted cautiously. Unless otherwise specified, statistical significance was set at *p* < 0.05.

## 5. Conclusions

Among the evaluated biomarkers, CASP1, TAS, TOS, and P2RX7 exhibited promising discriminatory performance in this case–control cohort, supporting their potential value for future biomarker research. However, because the present study evaluated only circulating protein concentrations, the observed alterations should be interpreted as changes in circulating pathway-related biomarkers rather than direct evidence of activation of the PANX1–P2RX7–NLRP3 signaling pathway or pyroptosis. Further functional validation studies and larger multicenter longitudinal investigations are warranted to clarify the biological significance and potential clinical utility of these biomarkers.

## 6. Limitations

This study has several limitations. First, its cross-sectional design precludes the establishment of causal relationships between alterations in circulating biomarkers and the pathophysiology of fibromyalgia syndrome. The exclusion of perimenopausal and postmenopausal women may limit the generalizability of the present findings to the entire female population with fibromyalgia. Second, only circulating protein concentrations were measured, which may not fully reflect molecular events occurring within immune cells, peripheral tissues, or the central nervous system. Third, the study did not include functional assays, such as ATP release, caspase-1 enzymatic activity, ASC speck formation, gasdermin cleavage, or pore formation. Therefore, the observed alterations should be interpreted as changes in circulating pathway-related biomarkers rather than direct evidence of activation of the PANX1–P2RX7–NLRP3 signaling pathway or pyroptosis. Fourth, the biomarker profile did not demonstrate coordinated alterations across all components of the canonical inflammasome pathway, indicating that alternative regulatory mechanisms or compartment-specific inflammatory responses may also contribute to the observed findings. Finally, this was a single-center case–control study, and the diagnostic performance of the evaluated biomarkers was not externally validated. Therefore, larger multicenter, longitudinal, and functional validation studies are required to confirm the biological and clinical significance of these findings.

## Figures and Tables

**Table 1:** Analysis of sociodemographic and clinical parameters in the patient and control groups.

Demographic/Clinical Characteristic	Case (n = 93)	Control (n = 93)	*p* Value
Female	91 (97.8%)	92 (98.9%)	*1.000 ^a^*
Male	2 (2.2%)	1 (1.1%)	
Age (years), mean ± SD	44.67 ± 10.44	42.85 ± 6.98	*0.165 ^b^*
Occupation: housewife	82 (88.2%)	77 (82.8%)	*0.245 ^c^*
Occupation: nurse	2 (2.2%)	3 (3.2%)	
Occupation: student	2 (2.2%)	0 (0.0%)	
Occupation: other employees	7 (7.5%)	13 (14.0%)	
Sleep disturbance, yes	75 (80.6%)	42 (45.2%)	<0.001 *^c^
Fatigue, yes	91 (97.8%)	72 (77.4%)	<0.001 *^c^
Headache, yes	78 (83.9%)	53 (57.0%)	<0.001 *^c^
Morning fatigue, yes	86 (92.5%)	49 (52.7%)	<0.001 *^c^
Dry mouth, yes	73 (78.5%)	45 (48.4%)	<0.001 *^c^
Leg numbness, yes	72 (77.4%)	42 (45.2%)	<0.001 *^c^
Dry eyes, yes	42 (45.2%)	25 (26.9%)	0.009 *^c^
Concentration difficulty, yes	82 (88.2%)	42 (45.2%)	<0.001 *^c^
Soft tissue swelling sensation, yes	59 (63.4%)	41 (44.1%)	0.008 *^c^
Family history of fibromyalgia, yes	33 (35.5%)	0 (0.0%)	<0.001 *^c^

Values are presented as n (%) unless otherwise stated. ^a^ Fisher’s exact test. ^b^ Independent-samples *t*-test. ^c^ Pearson chi-square test. * Statistically significant *p* < 0.05.

**Table 2:** Differences in protein levels across patient and control groups.

Protein	Group	Min	Max	Median (Q1–Q3)	*p* Value
CASP1 (ng/mL)	Case	0.67	17.93	5.84 (3.06–10.15)	<0.001 ^a^*
	Control	0.56	5.41	1.19 (0.86–2.21)	
GSDMD (ng/mL)	Case	0.75	7.28	1.59 (0.98–1.90)	<0.001 ^a^*
	Control	0.48	4.34	1.74 (1.43–2.13)	
GSDME (ng/mL)	Case	0.64	7.86	2.28 (1.90–3.42)	<0.001 ^a^*
	Control	0.84	5.81	1.62 (1.39–2.13)	
IL1-β (pg/L)	Case	365.53	1390.20	794.20 (674.87–874.53)	*0.248 ^a^*
	Control	406.87	2562.20	804.20 (669.87–1038.20)	
PANX1 (ng/mL)	Case	0.73	6.83	1.45 (1.26–3.87)	<0.001 ^a^*
	Control	0.91	2.52	1.29 (1.13–1.51)	
P2RX7 (ng/L)	Case	135.71	1366.09	1092.50 (968.97–1164.23)	<0.001 ^a^*
	Control	89.04	1435.32	564.55 (203.33–1057.76)	
NLRP3 (pg/mL)	Case	37.10	242.72	81.80 (69.39–110.14)	0.003 ^a^*
	Control	16.25	344.09	115.46 (89.61–128.41)	
IL-18 (ng/L)	Case	5.66	31.55	11.10 (9.29–14.53)	<0.001 ^a^*
	Control	3.26	50.26	16.57 (12.94–20.50)	
TAS (mmol Trolox equivalent/L)	Case	0.07	3.73	1.41 (0.95–1.82)	<0.001 ^a^*
	Control	2.40	4.04	3.01 (2.89–3.17)	
TOS (µmol H_2_O_2_ equivalent/L)	Case	0.51	87.81	69.47 (56.26–75.19)	<0.001 ^a^*
	Control	3.86	69.67	39.89 (27.57–51.40)	

^a^ Mann–Whitney U test. Values are presented as the median (Q1–Q3) and minimum–maximum values. * Statistically significant at *p* < 0.05.

**Table 3:** ROC curve analysis of serum biomarkers for discriminating patients with FMS from controls.

Protein	AUC (95% CI)	*p* Value	Cut-Off Point	Sensitivity (%)	Specificity (%)
CASP1 (ng/mL)	0.898 (0.851–0.944)	<0.001 *	3.96	72.0	97.8
GSDMD (ng/mL)	0.655 (0.576–0.734)	<0.001 *	1.18	43.0	92.5
GSDME (ng/mL)	0.754 (0.683–0.824)	<0.001 *	1.73	84.9	63.4
IL1B (pg/L)	0.549 (0.465–0.633)	*0.248*	928.53	84.9	34.4
PANX1 (ng/mL)	0.704 (0.629–0.779)	<0.001 *	2.53	44.1	100.0
P2RX7 (ng/L)	0.757 (0.684–0.831)	<0.001 *	792.69	92.5	60.2
NLRP3 (pg/mL)	0.626 (0.540–0.713)	0.003 *	87.20	61.3	79.6
IL18 (ng/L)	0.735 (0.660–0.811)	<0.001 *	12.46	64.5	84.9
TAS (mmol Trolox equivalent/L)	0.898 (0.842–0.954)	<0.001 *	2.36	83.9	100.0
TOS (µmol H_2_O_2_ equivalent/L)	0.849 (0.789–0.909)	<0.001 *	59.20	72.0	92.5

AUC: Area under the ROC curve; CI: Confidence interval; Statistically significant after Bonferroni correction (* *p* < 0.005).

**Table 4:** Binary logistic regression analysis of clinical parameters associated with FMS.

Variable	B	S.E.	*p* Value	OR [Exp(B)]	95% CI for OR
Sleep disturbance	0.793	0.437	*0.070*	2.210	0.938–5.209
Fatigue	1.322	0.923	*0.152*	3.750	0.614–22.910
Headache	1.034	0.469	0.028 *	2.813	1.121–7.056
Morning fatigue	1.536	0.544	0.005 *	4.644	1.598–13.499
Dry mouth	1.008	0.449	0.025 *	2.741	1.136–6.615
Leg numbness	1.066	0.429	0.013 *	2.902	1.251–6.732
Dry eyes	-0.146	0.440	*0.741*	0.864	0.365–2.049
Concentration difficulty	1.800	0.475	<0.001 *	6.052	2.386–15.349
Soft tissue swelling	0.779	0.422	*0.065*	2.179	0.953–4.981

Binary logistic regression. OR: odds ratio; CI: confidence interval. * Statistically significant *p* < 0.05.

**Table 5:** Binary logistic regression models according to biological pathways.

Model	Variable	B	SE	*p* Value	OR—Exp(B)	95% CI for Exp(B)
Purinergic signaling model	PANX1	1.092	0.250	<0.001 *	2.981	1.826–4.867
	P2RX7	0.003	0.001	<0.001 *	1.003	1.002–1.004
	Age	0.029	0.021	*0.163*	1.030	0.988–1.074
Inflammasome model	CASP1	0.816	0.135	<0.001 *	2.261	1.737–2.944
	NLRP3	−0.002	0.004	*0.604*	0.998	0.990–1.006
	IL18	−0.031	0.032	*0.334*	0.970	0.911–1.032
	Age	0.025	0.024	*0.305*	1.025	0.978–1.075
Pyroptosis executioner model	GSDMD	−0.819	0.284	0.004 *	0.441	0.253–0.769
	GSDME	0.715	0.163	<0.001 *	2.044	1.485–2.815
	Age	0.029	0.019	*0.120*	1.030	0.992–1.068
Oxidative stress model	TAS	−3.798	0.693	<0.001 *	0.022	0.006–0.087
	TOS	0.088	0.020	<0.001 *	1.092	1.050–1.136
	Age	0.040	0.038	*0.298*	1.041	0.965–1.122

Binary logistic regression. OR: odds ratio; CI: confidence interval. * Statistically significant *p* < 0.05.

**Table 6:** Spearman correlations between clinical characteristics and serum biomarkers in the overall study population.

Clinical Variable	CASP1 (ng/mL)	GSDMD (ng/mL)	GSDME (ng/mL)	IL1-β (pg/L)	PANX1 (ng/mL)	P2RX7 (ng/L)	NLRP3 (pg/mL)	IL-18 (ng/L)	TAS (mmol Trolox Equivalent/L)	TOS (µmol H_2_O_2_ Equivalent/L)
Age	0.104 (0.157)	−0.051 (0.488)	−0.038 (0.608)	−0.019 (0.792)	0.110 (0.137)	0.042 (0.569)	−0.089 (0.228)	−0.023 (0.756)	−0.034 (0.644)	0.009 (0.903)
Sleep disturbance	−0.212 (*0.004*) *	−0.040 (0.592)	−0.210 (*0.004*) *	0.063 (0.393)	−0.100 (0.175)	−0.152 (*0.039*) *	0.138 (0.060)	0.068 (0.355)	0.230 (*0.002*) *	−0.311 (*<0.001*) *
Fatigue	−0.149 (*0.043*) *	0.062 (0.401)	−0.181 (*0.013*) *	0.160 (*0.029*) *	0.010 (0.890)	−0.178 (*0.015*) *	−0.042 (0.571)	0.122 (0.096)	0.207 (*0.005*) *	−0.217 (*0.003*) *
Headache	−0.235 (*0.001*) *	−0.045 (0.541)	−0.035 (0.634)	0.036 (0.624)	−0.096 (0.192)	−0.142 (0.053)	−0.012 (0.874)	0.063 (0.390)	0.131 (0.074)	−0.120 (0.102)
Morning fatigue	−0.332 (*<0.001*) *	0.003 (0.968)	−0.185 (*0.011*) *	0.099 (0.180)	−0.091 (0.216)	−0.187 (*0.011*) *	0.126 (0.088)	0.189 (*0.010*) *	0.256 (*<0.001*) *	−0.339 (*<0.001*) *
Dry mouth	−0.213 (*0.004*) *	0.042 (0.571)	−0.102 (0.166)	0.150 (*0.041*) *	0.035 (0.638)	−0.162 (*0.027*) *	0.030 (0.682)	0.218 (*0.003*) *	0.208 (*0.004*) *	−0.231 (*0.001*) *
Leg numbness	−0.133 (0.070)	0.124 (0.093)	−0.138 (0.060)	0.090 (0.221)	0.033 (0.653)	−0.191 (*0.009*) *	0.110 (0.135)	0.153 (*0.037*) *	0.254 (*<0.001*) *	−0.207 (*0.005*) *
Dry eyes	−0.119 (0.106)	−0.013 (0.860)	−0.040 (0.592)	0.133 (0.071)	−0.089 (0.225)	−0.045 (0.546)	0.005 (0.948)	−0.033 (0.655)	0.131 (0.076)	−0.091 (0.219)
Concentration difficulty	−0.280 (*<0.001*) *	0.104 (0.157)	−0.162 (*0.027*) *	0.088 (0.234)	−0.038 (0.609)	−0.132 (0.072)	0.096 (0.191)	0.097 (0.187)	0.314 (*<0.001*) *	−0.353 (*<0.001*) *
Soft tissue swelling sensation	−0.175 (*0.017*) *	−0.147 (*0.045*) *	−0.049 (0.504)	−0.048 (0.512)	−0.063 (0.395)	−0.319 (*<0.001*) *	0.118 (0.108)	−0.036 (0.623)	0.042 (0.565)	−0.139 (0.058)
Family history of fibromyalgia	−0.327 (*<0.001*) *	0.228 (*0.002*) *	−0.150 (*0.041*) *	0.035 (0.634)	−0.165 (*0.025*) *	−0.183 (*0.013*) *	0.161 (*0.028*) *	0.167 (*0.023*) *	0.341 (*<0.001*) *	−0.280 (*<0.001*) *

Values are presented as Spearman’s correlation coefficient, r (two-tailed *p* value). Binary clinical variables were coded as 0 = absent/no and 1 = present/yes. Italic values and asterisks indicate statistically significant correlations. * *p* < 0.05.

**Table 7:** Serum biomarker levels according to self-reported dry eye and dry mouth symptoms among healthy controls.

Biomarker	Dry Eye Present (n = 25), Median (Min–Max)	Dry Eye Absent (n = 68), Median (Min–Max)	*p* Value	Dry Mouth Present (n = 45), Median (Min–Max)	Dry Mouth Absent (n = 48), Median (Min–Max)	*p* Value
CASP1	1.11 (0.63–3.03)	1.29 (0.56–5.41)	*0.176*	1.13 (0.56–3.92)	1.27 (0.59–5.41)	*0.536*
GSDMD	1.77 (1.09–2.92)	1.74 (0.48–4.34)	*0.808*	1.72 (0.51–4.34)	1.81 (0.48–4.27)	*0.475*
GSDME	1.64 (0.98–3.80)	1.62 (0.84–5.81)	*0.735*	1.60 (0.98–4.81)	1.63 (0.84–5.81)	*0.872*
IL1B	702.20 (406.87–1838.87)	826.53 (464.20–2562.20)	*0.050*	733.53 (464.20–2562.20)	843.53 (406.87–2226.20)	*0.358*
PANX1	1.21 (0.91–1.91)	1.31 (0.94–2.52)	*0.145*	1.26 (0.91–2.47)	1.39 (0.94–2.52)	*0.148*
P2RX7	426.99 (110.83–1334.94)	643.53 (89.04–1435.32)	*0.246*	548.53 (101.60–1399.81)	595.51 (89.04–1435.32)	*0.954*
NLRP3	114.61 (16.25–162.59)	115.66 (16.38–344.09)	*0.856*	114.75 (18.60–211.54)	115.89 (16.25–344.09)	*0.672*
IL18	16.62 (9.41–50.26)	16.46 (3.26–37.11)	*0.477*	14.82 (4.15–50.26)	16.97 (3.26–37.11)	*0.431*
TAS	2.95 (2.40–4.04)	3.03 (2.65–3.90)	*0.071*	2.99 (2.40–3.85)	3.02 (2.65–4.04)	*0.465*
TOS	43.40 (6.56–69.67)	38.82 (3.86–66.62)	*0.273*	37.13 (7.71–64.67)	43.76 (3.86–69.67)	*0.220*

Values are presented as the median (minimum–maximum). Comparisons were performed using the Mann–Whitney U test among healthy controls only. No statistically significant differences were observed between groups (all *p* ≥ 0.05).

## Data Availability

The original contributions presented in this study are included in the article. Further inquiries can be directed to the corresponding author.

## Abbreviations

The following abbreviations are used in this manuscript:

ATP	Adenosine Triphosphate
AUC	Area Under the Curve
CASP1	Caspase-1
CI	Confidence Interval
ELISA	Enzyme-Linked Immunosorbent Assay
FMS	Fibromyalgia Syndrome
GSDMD	Gasdermin D
GSDME	Gasdermin E
HRP	Horseradish Peroxidase
IL-1β	Interleukin-1 Beta
IL-18	Interleukin-18
NLRP3	NOD-, LRR- and Pyrin Domain-Containing Protein 3
NPV	Negative Predictive Value
PANX1	Pannexin-1
PPV	Positive Predictive Value
P2RX7	Purinergic Receptor P2X 7
ROC	Receiver Operating Characteristic
ROS	Reactive Oxygen Species
TAS	Total Antioxidant Status
TOS	Total Oxidant Status
TNF-α	Tumor Necrosis Factor Alpha
ACR	American College of Rheumatology
OR	Odds Ratio
SD	Standard Deviation
CV	Coefficient of Variation
SPSS	Statistical Package for the Social Sciences
CNS	Central Nervous System
